# Supplementary material for: Genetic interplay between transcription factor Pou4f1/Brn3a and neurotrophin receptor Ret in retinal ganglion cell type specification
Source: Neural Dev. 2021 Sep 21;16:5. doi: 10.1186/s13064-021-00155-z (PMC8454062; doi:10.1186/s13064-021-00155-z)
Supplement: Supplementary file 6 — Additional file 6: Supplementary Table 1. χ2 statistics for pair-wise comparisons between different sparse random recombination experimental groups (age and genotype) considering RGC subtype distribution [file 13064_2021_155_MOESM6_ESM.docx]

Supplementary Table 1. χ^2^ statistics for pair-wise comparisons between different sparse random recombination experimental groups (age and genotype) considering RGC subtype distribution

|  | E15 complete  Brn3a HET vs WT  (Fig.6, 7a) | E15 sparse  Brn3a HET vs KO  (Fig. 3, 5, 7b) | P0 sparse  Brn3a HET vs KO  (Fig. 3, 5, 7c) | P22 sparse  Brn3a HET vs KO  (Fig. 3, 5, 7d) | Sparse P22 vs P0  Brn3a HET  (Fig.7c vs. 7d, blue bars) | Sparse P22 vs E15  Brn3a HET  (Fig.7b vs. 7d, blue bars ) |
| --- | --- | --- | --- | --- | --- | --- |
| ChiStat | 10.29 | 48.08 | 37.92 | 14.45 | 10.52 | 175.22 |
| P value | 0.328 | 6*10^-7^ | 3.9*10^-5^ | 0.071 | 0.231 | 1.6*10^-30^ |
| Deg. of freedom | 9 | 10 | 10 | 8 | 8 | 13 |
| Total  Nr. Cells Cond1 | 169 | 324 | 89 | 148 | 148 | 148 |
| Total  Nr. Cells  Cond2 | 138 | 232 | 93 | 145 | 89 | 324 |
